# Vitamin D Intake and Status in 12-Month-Old Infants at 63–66° N

**DOI:** 10.3390/nu6031182

**Published:** 2014-03-21

**Authors:** Birna Thorisdottir, Ingibjorg Gunnarsdottir, Laufey Steingrimsdottir, Gestur I. Palsson, Inga Thorsdottir

**Affiliations:** 1 Unit for Nutrition Research, Landspitali University Hospital & Faculty of Food Science and Nutrition, School of Health Sciences, University of Iceland, Eiriksgata 29, IS-101 Reykjavik, Iceland; E-Mails: ingigun@landspitali.is (I.G.); laufey@hi.is (L.S.); ingathor@hi.is (I.T.); 2 Children’s Hospital, Landspitali University Hospital, Hringbraut, IS-101 Reykjavik, Iceland; E-Mail: gesturip@landspitali.is

**Keywords:** 25-hydroxyvitamin D, vitamin D, infant, dietary supplements, fortified foods

## Abstract

The objective was to assess the vitamin D status in healthy 12-month-old infants in relation to quantity and sources of dietary vitamin D, breastfeeding and seasons. Subjects were 76 12-month-old infants. Serum levels of 25-hydroxyvitamin D (25(OH)D) ≥ 50 nmol/L were considered indicative of vitamin D sufficiency and 25(OH)D < 27.5 nmol/L as being indicative of increased risk for rickets. Additionally, 25(OH)D > 125 nmol/L was considered possibly adversely high. Total vitamin D at 9–12 months (eight data collection days) included intake from diet and supplements. The mean ± SD of vitamin D intake was 8.8 ± 5.2 μg/day and serum 25(OH)D 98.1 ± 32.2 nmol/L (range 39.3–165.5). Ninety-two percent of infants were vitamin D sufficient and none at increased risk for rickets. The 26% infants using fortified products and supplements never/irregularly or in small amounts had lower 25(OH)D (76.8 ± 27.1 nmol/L) than the 22% using fortified products (100.0 ± 31.4 nmol/L), 18% using supplements (104.6 ± 37.0 nmol/L) and 33% using both (110.3 ± 26.6 nmol/L). Five of six infants with 25(OH)D < 50 nmol/L had no intake of supplements or fortified products from 0 to 12 months. Supplement use increased the odds of 25(OH)D > 125 nmol/L. Breastfeeding and season did not affect vitamin D status. The majority of infants were vitamin D sufficient. Our findings highlight the need for vitamin D supplements or fortified products all year round, regardless of breastfeeding.

## 1. Introduction

Vitamin D is a key nutrient for children’s well-being and growth, is essential for bone health [1] and may contribute to other health benefits [2]. Infant need for vitamin D can be met by synthesis in the skin when exposed to appropriate ultraviolet B wavelengths and by sufficient vitamin D intake, either from breast milk or other dietary sources [3]. At latitudes higher than ~50° N, little or no cutaneous vitamin D synthesis is possible during winter months [4]. Vitamin D from breast milk alone is unlikely to meet the needs of infants during complementary feeding [5]. Few common foods are naturally rich in vitamin D [6]. Vitamin D intake from supplements or fortified foods or beverages is therefore important in northern latitudes [3].

The recommended intake (RI) of vitamin D is 10 μg (400 IU) for infants and children from six months of age according to Nordic nutrition recommendations [7]. This is in accordance with the average intake specified by the Institute of Medicine (IOM) for infants from birth to 12 months of age [8]. To ensure that the RI is met, parents are advised to give their infants a daily supplement of 10 μg D_3_ from the age of 1–2 weeks. Fortification schemes differ between countries [3]. In Iceland, population-based studies on infants [9,10] and pre-school children [11] have shown that less than two-thirds of young children use vitamin D supplements regularly. Several cases of rickets in the past years have given cause for concern on the vitamin D status of Icelandic infants [12]. In 2003, a follow-up formula intended for infants from 6 to 24 months, fortified with 1.2 μg D_3_ per 100 mL, was introduced [13], and infant porridges and breakfast cereals fortified with vitamin D are available [14]. Population-based studies on both vitamin D intake and status during the complementary feeding period in the Nordic countries are lacking [3]. The vitamin D status of Icelandic infants is unknown and has never been studied.

The objectives of this study were to assess vitamin D status measured as serum levels of 25-hydroxyvitamin D (25(OH)D) in healthy 12-month-old infants and to consider it in relation to quantity and sources of dietary vitamin D, breastfeeding and seasons.

## 2. Experimental Section

### 2.1. Subjects

Study subjects were 76 infants with data on dietary intake in infancy and quantitative analysis of serum 25(OH)D levels at 12 months. They were a subsample of participants in a longitudinal cohort study on diet, growth and health outcomes of infants born in the year 2005. In the original study, 250 healthy Icelandic infants born at term were randomly selected from the whole country (63–66° N). Blood samples were obtained with the primary aim of analyzing the iron status and blood lipids of the infants [13]. Analysis of serum 25(OH)D was only possible for those subjects with sufficient amounts of blood available, resulting in the subsample of 76. Anthropometrical variables and dietary intake in infancy, e.g., duration of breastfeeding, intake of vitamin D, formula and cod liver oil, sociodemographic factors (parents’ age and education) and parental BMI of the children included in the current analysis did not differ from those of the children in the original study. More detailed information on the original cohort is published elsewhere [13,15]. Informed written consent from the parents was obtained, and all individual information was processed with strict confidentiality. The study was approved by the Icelandic Bioethics Committee, the Icelandic Data Protection Authority and the Local Ethical Committee at Landspitali University Hospital.

### 2.2. Dietary Assessment

**Figure 1:**
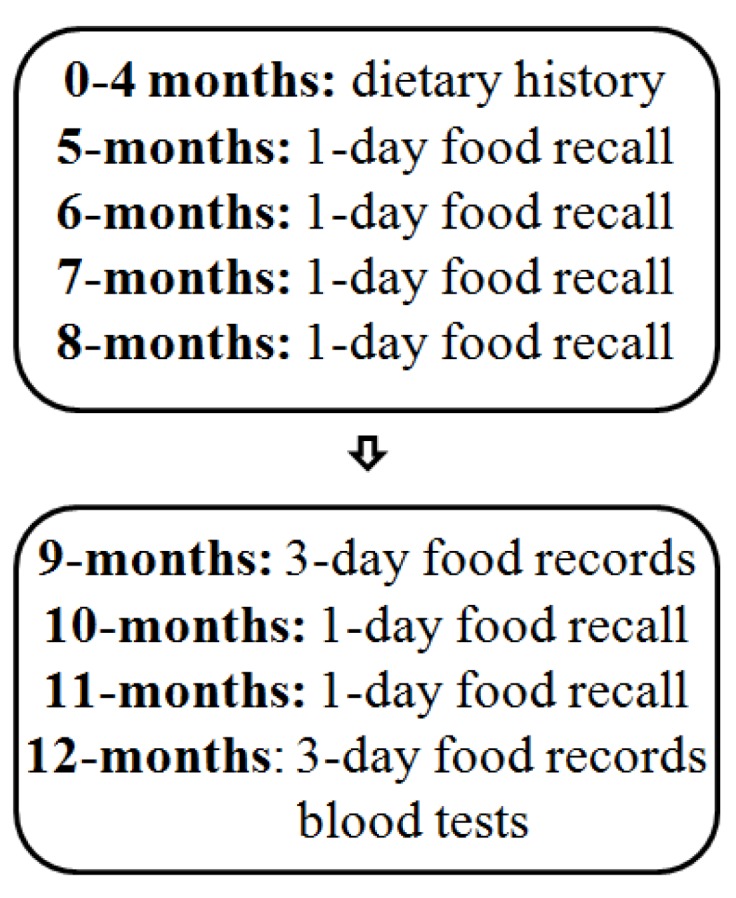
Flowchart on the progress of the study.

A flowchart of the process of the study, relevant to the present analysis, is presented in Figure 1.

Dietary data from 0 to 4 months of age were collected by dietary history, including questions on breastfeeding, infant formula-feeding, other food items and supplements. At 5–8 and 10–11 months, 24-h recalls using common household measures, such as cups and spoons, were made. At 9 and 12 months, weighed food records were kept for 3 consecutive days (72-h). All food and fluids were weighed on accurate scales (Philips type HR 2385, Szekesfehervar, Hungary) with 1-g precision. The amount of breast milk consumed was estimated by weighing the breastfed infants in the same clothes before and after each breastfeeding session on baby scales (Tanita model 1583, Tokyo, Japan, or Sega model 336, Hamburg, Germany) with 10-g precision. An average consumption of food and nutrients was calculated using the Icelandic food composition database [14]. The total intake of vitamin D included intake from the diet, breast milk and supplements. For the analysis presented here, the main emphasis was on dietary intake at 9–12 months, because we believe that it may influence serum 25(OH)D concentration at 12 months [3,16]. We divided infants into four groups based on regular intake of significant amounts of the main vitamin D sources at 9–12 months. The “fortified” group included infants getting on average ≥2.4 μg of vitamin D per day from fortified products; the “supplement” group included those getting on average ≥5.0 μg of vitamin D per day from supplements; the “combined” group included those fulfilling both conditions; and the “no or irregular” group included infants fulfilling neither conditions. Fortified products included infant formula, infant porridges and breakfast cereals, and the cut-off at 2.4 μg of vitamin D was applied, because it corresponds to consumption of ≥200 mL of fortified formula, the most commonly consumed product in this category. Supplements included cod liver oil and liquid vitamin A and D supplements (vitamin AD drops), and the cut-off at 5 μg of vitamin D was applied, because it corresponds to the recommended dose on at least half of the data collection days. We also considered whether or not infants were still partially breastfed at 12 months of age.

### 2.3. Blood Sampling and Biochemical Analyses

At 12 months of age, blood samples were collected in the morning in the fasting state. The samples were centrifuged within 6 h of data collection. Separated serum samples were then stored at −80 °C until being analyzed. The quantitative analyses of serum 25(OH)D levels were conducted by the Roche Diagnostics Vitamin D total assay (Roche Diagnostics, Mannheim, Germany), with a measuring range of 7.5–175 nmol/L and a precision of 0.1 nmol/L. In accordance with a recent Nordic systematic literature review (SLR), serum 25(OH)D ≥ 50 nmol/L (20 ng/mL) was considered indicative of a sufficient vitamin D status, and serum 25(OH)D < 27.5 nmol/L (11 ng/mL) indicates increased risk for rickets [3]. Additionally, serum 25(OH)D > 125 nmol/L (50 ng/mL) was considered as possibly adversely high, as suggested by the IOM [8]. Infants were classified according to season when blood samples were collected; winter/spring (January 2006–April 2006 and November 2006–December 2006) and summer/autumn (May 2006–October 2006).

### 2.4. Statistical Analyses

Statistical analyses were performed with SAS (Enterprise Guide 4.3; SAS Institute Inc., Cary, NC, USA). Linear regression analysis was used to examine the relation between vitamin D intake and serum 25(OH)D. Descriptive statistics were used to describe vitamin D intake and serum 25(OH)D concentrations, presented as the means ± SD. For comparison between groups, an independent, two-sample *t*-test with equal variances and a one-way ANOVA with equal variance were used. Logistic regression was used to examine the risk of having serum 25(OH)D above 125 nmol/L among infants using supplements or not. The results were presented as odds ratios (OR), with its 95% CI. Spearman’s correlation analysis was used to assess correlations between 25(OH)D and breastfeeding, presented as the correlation coefficient (ρ) and the *p*-value for correlation. A two-sided test with a *p*-value < 0.05 was considered statistically significant.

## 3. Results

At the age of 12 months, the mean ± SD serum 25(OH)D concentration was 98.1 ± 32.2 nmol/L (39.3 ± 12.9 ng/mL) and ranged from 39.3 to 165.5 nmol/L (15.7 to 66.3 ng/mL). Seventy infants (92%) were considered vitamin D sufficient and none at increased risk for rickets. Eighteen infants (24%) were considered to have a possibly adversely high 25(OH)D concentration.

**Figure 2:**
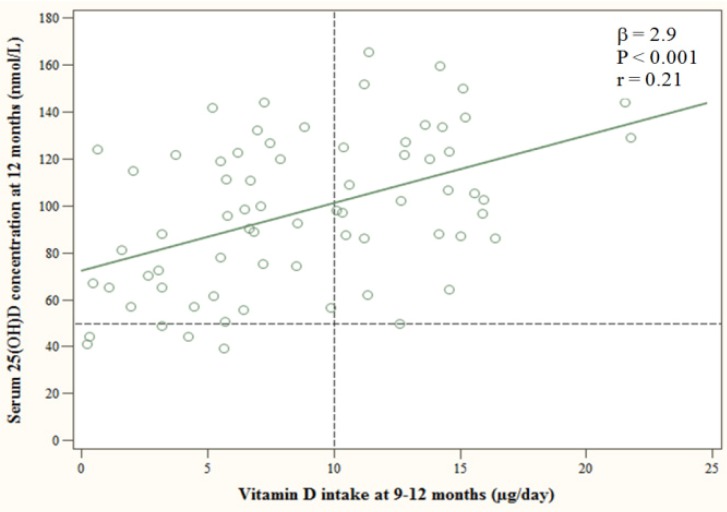
The linear regression line for serum 25-hydroxyvitamin D (25(OH)D) at 12 months in relation to vitamin D intake from diet and supplements at 9–12 months. The dashed horizontal line at 50 nmol/L is the cut-off line applied for a sufficient vitamin D status, and the dashed vertical line at 10 μg indicates the Nordic recommended intake (RI).

Vitamin D intake at 9–12 months predicted 25(OH)D levels at 12 months (Figure 2). The mean ± SD intake of vitamin D was 8.8 ± 5.2 μg, and 57% of the infants were below the RI of 10 μg. Those infants had significantly lower mean ± SD 25(OH)D than infants above the RI (87.1 ± 31.1 *vs*. 111.8 ± 29.0 nmol/L, *p* = 0.001).

Supplements provided 56% of total vitamin D at 9–12 months. Another 38% came from fortified products; thereof, 24% from formulas, 13% from infant porridges and 1% from breakfast cereals. Among natural sources of vitamin D were meat and fish (3%) and cow’s milk (1%). Breast milk provided <1% of vitamin D. As presented in Table 1, infants in the “combined” group had a higher vitamin D intake than infants in the “supplement” group (*p* < 0.001), who, in turn, had a higher vitamin D intake than infants in the “fortified” group (*p* = 0.013). Mean serum 25(OH)D in these three groups was, however, not significantly different (*p* > 0.05). Infants not using fortified products or supplements regularly in significant amounts at 9–12 months (“no or irregular” group) had significantly lower vitamin D intake than all the other groups (*p* < 0.001) and lower serum 25(OH)D (*p* < 0.001).

**Table 1:** Variables potentially associated with vitamin D intake at 9–12 months and serum 25(OH)D at 12 months.

Variables	*n* (%)	Vitamin D Intake (μg/day)	25(OH)D (nmol/L)
All	76 (100)	8.8 ± 5.2	98.1 ± 32.2
Boys	39 (51)	8.6 ± 5.7	96.6 ± 34.3
Girls	37 (49)	8.9 ± 4.6	99.7 ± 30.3
*Vitamin D sources at 9–12 months* ^a^			
“No or irregular”	20 (26)	2.5 ± 1.9	76.8 ± 27.1
“Fortified”	17 (22)	6.5 ± 2.2	100.0 ± 31.4
“Supplement”	14 (18)	8.8 ± 2.7	104.6 ± 37.0
“Combined”	25 (33)	14.3 ± 3.0	110.3 ± 26.6
*Partially breastfed at 12 months*			
No	62 (82)	8.7 ± 5.0	97.7 ± 32.7
Yes	14 (18)	9.1 ± 6.0	101.9 ± 31.5
*Season of blood sample collection*			
Winter/Spring	33 (43)	8.1 ± 4.9	94.4 ± 31.6
Summer/Autumn	43 (57)	9.2 ± 5.4	101.0 ± 32.8

Abbreviation: 25(OH)D, 25-hydroxyvitamin D. Mean ± SD. ^a^ Infants were divided into groups based on the regular intake of significant amounts of the main vitamin D sources at 9–12 months. “No or irregular”: neither fortified products nor supplements; “Fortified”: fortified products; “Supplement”: supplements; “Combined”: both fortified products and supplements.

Five out of six infants with serum 25(OH)D below 50 nmol/L belonged to the “no or irregular” group, *i.e.*, they did not use fortified products or supplements on a regular basis or in significant amounts at 9–12 months. Their intake of vitamin D was below 5 μg at 9–12 months. Further, they did not use fortified products or supplements at all from birth to nine months of age. The sixth infant with 25(OH)D below 50 nmol/L was categorized in the “supplement” group, but only got half of the recommended amount of supplements daily. Of the 18 infants with 25(OH)D levels above 125 nmol/L, one belonged to the “no or irregular” group (5% of infants in that group), three to the “fortified” group (18%), six to the “supplement” group (43%) and eight to the “combined” group (32%). Infants using supplements (*i.e.*, classified in the “supplement” or “combined” groups) were 4.6 times more likely (95% CI = 1.4, 15.8) to have 25(OH)D above 125 nmol/L than infants not using supplements (*i.e.*, classified in the “no or irregular” or “fortified” groups). Infants in the “combined” group were not more likely to have 25(OH)D above 125 mol/L than infants in the “supplement” group (OR (95% CI) = 0.6 (0.2, 2.4)).

The duration of exclusive breastfeeding ranged from 0 to 6 months, with a median (25th, 75th percentiles) of four (1, 5) months. The total duration of breastfeeding ranged from 0 to 12 months, with a median (25th, 75th percentiles) of eight (6, 10) months. There was no correlation between the duration of exclusive breastfeeding and 25(OH)D (ρ = −0.02, *p* = 0.895) or the total duration of breastfeeding and 25(OH)D (ρ = 0.08, *p* = 0.502). Among children partially breastfed at 12 months of age, breast milk intake in the age period of 9–12 months ranged from 10 mL to 750 mL per day. No difference was observed in vitamin D intake or 25(OH)D according to breastfeeding at 12 months (*p* = 0.923 and 0.674, respectively), season of blood sample collection (*p* = 0.385 and *p* = 0.379, respectively) or sex (*p* = 0.859 and *p* = 0.678, respectively).

## 4. Discussion

This study provides the first information on vitamin D status in Icelandic infants. Based on thresholds proposed in a recent Nordic SLR [3], 92% of the infants were considered vitamin D sufficient and none at increased risk for rickets. Consensus has not been reached on the optimal 25(OH)D concentration in infants, and uniformity is lacking in the description of sufficient and deficient ranges for 25(OH)D levels. Using cut-off values proposed by IOM [8] or the Pediatric Endocrine Society [16] does not change our results of 92% of infants being classified as vitamin D sufficient, and according to those cut-offs, no infants are classified as vitamin D deficient. According to a European consensus statement, vitamin D deficiency occurs commonly among healthy European infants not adhering to recommendations for vitamin D supplementation [17]. However, studies on healthy infants from Denmark [18], Norway [19] and Finland [20] have previously reported a high proportion of vitamin D sufficiency amongst nine-month-olds, 12-month-olds and 14-month-olds, respectively. Those studies were not population-based, and in the Danish and Finnish studies, selection bias resulted in an unusually high frequency of infants using vitamin D supplements (97% and 100%, respectively). The Nordic countries have a well-established newborn and infant healthcare. According to protocols for the newborn and infant healthcare in Iceland [21], mothers are asked about their infants’ vitamin D supplement use and encouraged to follow the recommendations on vitamin D supplements at every visit, which are scheduled at least nine times during the first year of the infant. This may explain the relatively low proportion of vitamin D deficiency among infants in Iceland and, more broadly, the Nordic countries. To our knowledge, this is the first study on infant vitamin D status in the Nordic countries in a sample that is representative of the general infant population. Therefore, we believe it is an important contribution to the literature on the vitamin D status of healthy infants during complementary feeding in northern latitudes.

The relatively high 25(OH)D levels may, at least partly, be explained by 75% of the infants regularly using vitamin D supplements and/or fortified foods or drinks in significant amounts. The commonly used follow-up formula, fortified with vitamin D, was introduced in 2003. Before that, it was common that regular cow’s milk gradually replaced breast milk in the age range of 5–12 months [9]. The main vitamin D source for Icelandic infants has, therefore, historically, been vitamin D drops or cod liver oil, and even though studies on infants and children have shown a little less than two-thirds of children complying with supplement use, the remaining one-third has been seen as a reason for concern. Studies on Icelandic infants and young children have never before assessed how frequently vitamin D fortified products are consumed or how they contribute to vitamin D status. We do not have any data on the vitamin D status of infants and young children previous to the introduction of the fortified follow-up formula.

The wide range of serum 25(OH)D concentrations observed in the study should be considered when interpreting the results. Transferring the 8% of infants in our sample with serum 25(OH)D below 50 nmol/L to the whole infant population in Iceland (around 4600 12-month-olds annually from 2005 to 2012) [22] suggests that about 275 infants every year would be vitamin D insufficient, with the possibility of some being at risk for vitamin D deficiency. Our study, showing that infants with an insufficient vitamin D status did not use fortified products or supplements at all from birth to nine months of age, in addition to a very low vitamin D intake from nine to 12 months of age, could be considered in newborn and infant healthcare in Iceland to identify, at an early age, children with undesirable diet habits that may increase the risk of vitamin D insufficiency or deficiency. Infants using supplements with or without concurrent use of fortified foods or drinks were more likely than infants not using supplements to have 25(OH)D concentrations that may be considered as possibly adversely high [8]. Correct dosing of supplements is important, as well as caution when combining the use of supplements and fortified foods or drinks. However, no infant exceeded the 25 μg vitamin D intake, which is considered the tolerable upper intake level by the European Food Safety Authority [6], and other estimations of the high end for safe concentration levels of 25(OH)D are higher than the 125 nmol/L estimated by IOM [16,23].

Since the time of this study, parents have been encouraged to give their infants vitamin D drops instead of vitamin AD drops. The vitamin D content in the two products is the same, and other infant guidelines remain unchanged. Therefore, we believe that the findings of this study are transferable to Icelandic infants born today. Iceland is among the few countries that includes cod liver oil intake or other vitamin D supplements in the population-based dietary guidelines for children and adults of all ages [24]. A recent study from Denmark showed that parents’ perceived relevance of nutritional guidelines declined from the early to later phases of complementary feeding [25], and a Finnish study showed decreased use of supplements as children grew older [26]. Icelandic studies have also shown low vitamin D intakes among children [27], adolescents [28] and adults [29], and results from a follow-up of the infants participating in the current analysis reveal that only 27% used supplements at six-years of age [30]. While the vitamin D status in our study is considered sufficient for the majority of infants, studies on Icelandic children and adults have shown lower 25(OH)D concentrations than presented here [31,32,33]. This study, showing the importance of supplements and/or fortified products on vitamin D status, is therefore of importance for public health policy.

We did not find differences in 25(OH)D levels between months when cutaneous synthesis is expected to be very low or totally absent at northern latitudes (November to April) and months when the quantity and quality of UV radiation might be sufficient for cutaneous synthesis (May to October). Icelandic parents are advised to keep their infants out of direct sunlight, and summer temperatures in Iceland usually require long sleeves and a hat for infants. In case infants get in contact with sun, the use of sunscreen is advised [21]. We propose that cutaneous synthesis of vitamin D does not contribute significantly to 25(OH)D in Icelandic infants and that the use of supplements and/or fortified foods and drinks is therefore essential all year round in order to maintain a sufficient vitamin D status. Seasons have, however, been shown to affect 25(OH)D in older children and adults [31,33]. No difference was seen in 25(OH)D levels between infants breastfed or not breastfed at 12 months, which may be explained by the emphasis put on supplement use regardless of feeding mode.

The main strengths of our study lie in the assessment of both vitamin D intake and status in a population-based infant sample and the longitudinal design of the study. Although we are aware of the possibility of altered dietary behavior on data collection days, the use of eight data collection days from 9 to 12 months of age in the current analysis strengthens our confidence that we have reliably estimated food and nutrient intake that may affect vitamin D status at 12 months. The dietary information from 0 to 8 months of age gives practical information. The study also has some limitations. Blood samples were obtained with the primary aim of analyzing the iron status and blood lipids of the infants [13]. Analysis of serum 25(OH)D was only possible for those subjects with sufficient amounts of blood available, resulting in a small sample size. Analyses on parameters that have been used to complement 25(OH)D levels and/or used as blood safety measurements in other studies [34,35], such as parathyroid hormone, serum calcium, alkaline phosphatase and C-reactive protein, were not performed. There is a possibility that the method used for quantitative analyses of serum 25(OH)D may overestimate the 25(OH)D concentration in infants, due to the possible presence of *C*-3 epimers [36,37,38]. As all subjects were of Icelandic origin and healthy, transferring the results to high-risk groups of vitamin D deficiency, such as infants of non-western immigrants residing in northern latitudes and infants with chronic illnesses, should not be advised [7,39,40].

## 5. Conclusions

In conclusion, the majority of infants were vitamin D sufficient. Our findings highlight the need for vitamin D supplements or fortified products all year round, regardless of breastfeeding in infant populations with little or no sun exposure.
